# 硅胶基质固定相离子对反相液相色谱法测定强离解化合物的正辛醇/水分配系数

**DOI:** 10.3724/SP.J.1123.2021.02005

**Published:** 2021-11-08

**Authors:** Xiaolan LIU, Wei GAO, Chao LIANG, Junqin QIAO, Kang WANG, Hongzhen LIAN

**Affiliations:** 1.生命分析化学国家重点实验室, 南京大学化学化工学院, 南京大学现代分析中心, 江苏 南京 210023; 1. State Key Laboratory of Analytical Chemistry for Life Science, School of Chemistry & Chemical Engineering and Center of Materials Analysis, Nanjing University, Nanjing 210023, China; 2.济川药业集团有限公司, 江苏 泰兴 225441; 2. Jumpcan Pharmaceutical Group Co., Ltd., Taixing 225441, China

**Keywords:** 离子对反相液相色谱, 强离解化合物, 正辛醇/水分配系数, 定量结构-保留行为关系, 硅胶基质色谱柱, ion-pair reversed-phase liquid chromatography (IP-RPLC), strong ionized compounds, *n*-octanol/water partition coefficient (log *P*), quantitative structure-retention relationship (QSRR), silica-based column

## Abstract

反相液相色谱(RPLC)是测定正辛醇/水分配系数(log *P*)的有效方法,但由于缺少同类型模型化合物,RPLC在测定强离解化合物的log *P*时遇到挑战。该文在硅胶基质C18色谱柱上,采用离子抑制反相液相色谱(IS-RPLC)和离子对反相液相色谱(IP-RPLC)分别对中性化合物、酚酸、羧酸、磺酸及部分两性化合物的保留行为进行了系统研究。在IS-RPLC模式下,利用中性化合物、弱离解的酚酸和苯羧酸作为模型化合物,建立了表观正辛醇/水分配系数(log *D*)与纯水相保留因子对数值(log *k*_w_)的定量结构-保留行为关系(QSRR)模型,测定了19种离解化合物的log *D*值,作为后续IP-RPLC的模型化合物及验证化合物。在IP-RPLC模式下,将中性、弱离解和强离解化合物作为混合模型组,以溶质静电荷*n*_e_、氢键酸碱性参数*A*和*B*为桥梁,建立了线性良好的log *D*-log *k*_w-IP_模型,采用3种不同类型的离解化合物进行了外部验证实验,预测值误差低于10%,证实了模型的可靠性。在此基础上,预测了8种强离解化合物的log *D*_7.0_值(pH 7.0条件下的log *D*值)。研究表明,利用结构相关参数沟通不同类型的模型化合物,是实现IP-RPLC测定强离解化合物log *D*值的一种行之有效的方法。与聚乙烯醇基质色谱柱相比,通用型的硅胶基质色谱柱上尽管存在着更多的次级作用,但可以为强离解化合物log *D*的测定提供更灵活的选择。

## Abstract (en)

The *n*-octanol/water partition coefficient (log *P*) is an important parameter to characterize the overall hydrophobicity of organic compounds. Reversed-phase liquid chromatography (RPLC) has been recommended as an effective method for the indirect determination of log *P* by the Organization for Economic Cooperation and Development (OECD). Using RPLC, most studies focus on the determination of log *P* or the apparent *n*-octanol/water partition coefficient (log *D*) of neutral compounds and weakly ionized compounds. However, the experimental log *P* or log *D* values of strongly ionized compounds have rarely been reported. In our previous work, the experimental log *D* of strongly ionized compounds could be determined well by ion-pair reversed-phase liquid chromatography (IP-RPLC) on an octadecyl-poly(vinyl alcohol) column using the log *D*-log *k*_w-IP_ model established by different types of model compounds. However, the universality of this strategy for different chromatographic columns has yet to be verified. In this study, the retention behavior of neutral compounds, phenolic acids, carboxylic acids, sulfonic acids, and some amphoteric compounds was systematically investigated on a silica-based C18 column (150 mm×4.6 mm, 5 μm) via ion-suppressed RPLC (IS-RPLC) and IP-RPLC, respectively. In the IS-RPLC mode, methanol and 20 mmol/L ammonium dihydrogen phosphate buffer (pH 7.0) were used as the mobile phase to perform isocratic elution at different methanol ratios. The log *k*_w_ values of the test compounds were obtained using the linear solvent strength (LSS) model. Neutral compounds, weakly ionized phenolic acids and benzene carboxylic acids were then used as model compounds to establish the log *D*-log *k*_w-IS_ model. The quantitative structure-retention relationship (QSRR) model, including structure-related descriptors like the charge (*n*_e_) and Abraham solvation parameters (*A* and *B*), exhibited much better correlation than the unary linear regression model between log *D* and log *k*_w-IS_. The log *D*_7.0_ (log *D* under pH 7.0) values of 19 ionized compounds were then determined by the model; the determined compounds were used as model compounds and validation compounds in IP-RPLC. In the IP-RPLC mode, besides methanol and ammonium dihydrogen phosphate buffer, the mobile phase also contained tetrabutylammonium bromide, as an ion-pair reagent. The retention behaviors of all tested compounds conformed well with the LSS model even under IP-RPLC, with a log *k*-*φ* linear correlation coefficient (*R* ^2^) greater than 0.99. The log *D*-log *k*_w-IP_ model was then established using 62 compounds as a mixed model set, including neutral, weakly ionized, and strongly ionized compounds. Similarly, by introducing *n*_e_, *A*, and *B*, the log *D*-log *k*_w-IP_ model showed good linearity, with *R* ^2^ greater than 0.94. Comparing the log *D*-log *k*_w-IP_ model established on the silica-based C18 column in this work with that established on a poly(vinyl alcohol)-based C18 column in our previous work, *n*_e_, *A*, and *B* contributed more to the model in this work, indicating there was a greater secondary effect on the silica-based column. To confirm the reliability of the log *D*-log *k*_w-IP_ model, three different types of acidic compounds were used as validation compounds. The predicted log *D* of the three ionic compounds was very similar to that determined by the shake-flask method (SFM)/slow stirring method (SSM) or IS-RPLC method in this work, confirming the reliability of the model. Based on the above results, the log *D*_7.0_ values of eight strongly ionized compounds were predicted by IP-RPLC. The findings suggested that IP-RPLC is a promising method to predict the experimental log *D* of strongly ionized compounds, and that the conventional silica-based C18 column offers more flexible options in log *D* determination.

正辛醇-水分配系数(log *P*)是化合物亲酯性/亲水性的量度,是表征化合物整体疏水性的重要参数,可用于研究有机小分子和生物体之间的相互作用,在指导药物合理设计及毒性机理研究^[1]^、环境风险评价^[2,3]^以及生物降解研究^[4]^中具有重要作用。log *P*测定的经典方法为摇瓶法(SFM)^[5]^和慢搅法(SSM)^[6]^,但测试需要大量样品,成本很高且费时。反相液相色谱法(RPLC)是经济合作与发展组织(OECD)推荐的间接测定log *P*的有效方法,它是利用与待测物质结构相似且已有准确可靠log *P*值的化合物作为模型化合物,通过测得特定条件下模型化合物的保留因子对数值log *k*,建立log *P*与log *k*之间的定量结构-保留行为关系(QSRR)模型,进而求得被测化合物的log *P*值^[7]^。log *P*对应化合物中性状态的疏水性,对于离解性化合物,通常采用表观正辛醇-水分配系数(log *D*)来表征物质在不同pH条件下的疏水性。研究发现,与log *P*-log *k*模型相比,以100%水相作流动相时的保留因子对数值(log *k*_w_)与log *P*或log *D*之间具有更好的相关性,即Collander方程^[8]^。目前,利用RPLC研究中性化合物的log *P*^[9]^以及利用离子抑制反相液相色谱法(IS-RPLC)研究弱酸或弱碱化合物的log *P*或log *D*已经比较成熟^[10,11]^。然而,对于强离解化合物,使用IS-RPLC法分析时,即使在极低比例的有机调节剂和极端pH条件下,也很难得到有效保留。

离子对反相液相色谱(IP-RPLC)作为IS-RPLC的补充,通过在流动相中添加离子对试剂增强溶质的保留,专门用于强离解化合物的分离分析^[12,13,14,15]^。但有实验log *P*或log *D*值的强离解化合物很少,不利于QSRR模型的建立,目前,IP-RPLC用于强离解化合物log *P*或log *D*的研究还非常少。20世纪90年代,邹汉法等^[16]^利用IP-RPLC以苯磺酸作为模型化合物,建立了log *D*-log *k*_w_预测模型,但模型化合物log *D*的获取均来源于计算方法,因此,对于log *D*实验值预测的准确性不能保障。最近,本课题组Liang等^[17]^在聚乙烯醇基质C18柱上,以溶质静电荷(*n*_e_)、氢键酸性参数(*A*)和氢键碱性参数(*B*)沟通了有实验log *D*值的中性化合物、弱酸和强酸化合物,作为混合模型组,建立了更为可靠的log *D*预测模型,为强离解化合物log *D*实验值的获取提供了研究基础。

然而,聚乙烯醇基质色谱柱的柱效和寿命均比传统硅胶基质色谱柱低,为了考察Liang等^[17]^提出的测定强离解化合物log *D*模型的通用性,本文采用最常规的硅胶基质C18柱,通过扩大模型化合物的范围,以磷酸二氢铵为缓冲盐,四丁基溴化铵作为离子对试剂,甲醇为有机调节剂,使用等度洗脱的方法获取了26种中性化合物、47种离解化合物分别在IS-RPLC和IP-RPLC模式下的log *k*_w_值,建立了相应的log *D*-log *k*_w_ QSRR模型。

## 1 实验部分

### 1.1 药品与试剂

实验中所用色谱级甲醇购自Honeywell公司(USA),所用水均为饮用纯净水(杭州娃哈哈集团);分析纯磷酸二氢铵、氨水(25%~28%)和磷酸(85%)均购自南京化学试剂股份有限公司,分析纯四丁基溴化铵(99%)购自北京百灵威科技有限公司。

实验中所用化合物列于表1, log *P*和p*K*_a_值为ACD/Labs软件数据库模块中的文献实验值,溶质静电荷*n*_e_、氢键酸碱性参数*A*、*B*由https://ilab.acdlabs.com/iLab2/获取。这些化合物分别购自Accu Standard (USA)、TCI(Japan)、国药集团化学试剂有限公司(上海)、Acros Organics(USA)、Matrix Scientific (USA)和Sigma-Aldrich (USA),所有物质的纯度均大于98%。

**表1:** 实验用化合物的log *P*、 p*K*_a_、 log *D*_7.0_、 log *k*_w-IS_、 log *k*_w-IP_、 *n*_e_、 *A*和*B*值

Compound type		No.			Compound		log P		pK_a_		log D_7.0_		log k_w-IS_		log k_w-IP_		n_e_		A		B
Neutral			N1			benzyl alcohol	1.10		NA		1.10		1.32		1.32		0.00		0.39		0.56
			N2			1,4-xylene	3.15		NA		3.15		3.23		3.23		0.00		0.00		0.16
			N3			toluene	2.73		NA		2.73		2.66		2.66		0.00		0.00		0.14
			N4			ethylbenzene	3.15		NA		3.15		3.20		3.20		0.00		0.00		0.15
			N5			anisole	2.11		NA		2.11		5.60		5.60		0.00		0.00		0.29
			N6			benzyl chloride	5.50		NA		5.50		2.64		2.64		0.00		0.00		0.14
			N7			hexamethyl-benzene	4.61		NA		4.61		4.65		4.65		0.00		0.00		0.12
			N8			pentachlorobenzene	5.18		NA		5.18		4.84		4.84		0.00		0.00		0.00
			N9			1,2-xylene	3.12		NA		3.12		3.09		3.09		0.00		0.00		0.16
			N10			biphenyl	4.01		NA		4.01		3.75		3.75		0.00		0.00		0.26
			N11			phenyl ether	4.21		NA		4.21		3.73		3.73		0.00		0.00		0.19
			N12			naphthalene	3.30		NA		3.30		3.13		3.13		0.00		0.00		0.20
			N13			2-methylnaphthalene	3.86		NA		3.86		3.65		3.65		0.00		0.00		0.20
			N14			1,3-dichlorobenzene	3.53		NA		3.53		3.32		3.32		0.00		0.00		0.02
			N15			tribromobenzene	4.51		NA		4.51		4.34		4.34		0.00		0.00		0.00
			N16			chlorobenzene	2.89		NA		2.89		2.72		2.72		0.00		0.00		0.07
			N17			bromobenzene	2.99		NA		2.99		2.84		2.84		0.00		0.00		0.09
			N18			benzhydrol	2.67		NA		2.67		2.86		2.86		0.00		0.41		0.77
			N19			pentabromotoluene	5.87		NA		5.87		5.62		5.62		0.00		0.00		0.00
			N20			hexabromobenzene	6.80		NA		6.80		5.63		5.63		0.00		0.00		0.00
			N21			benzaldehyde	1.47		NA		1.47		1.33		1.33		0.00		0.00		0.39
			N22			1,3-xylene	3.20		NA		3.20		3.19		3.19		0.00		0.00		0.12
			N23			benzene	2.22		NA		2.22		2.08		2.08		0.00		0.00		0.14
			N24			1,4-diacetoxybenzene	0.98		NA		0.98		1.45		1.45		0.00		0.00		0.77
			N25			1,4-dibromonaphthalene	5.02		NA		5.02		4.51		4.51		0.00		0.00		0.16
			N26			1-bromonaphthalene	4.22		NA		4.22		3.77		3.77		0.00		0.00		0.13
Carboxylic acids and			W1			benzoic acid	1.87		4.20		-0.93		0.43		1.68		-1.00		0.59		0.40
phenolic acids			W2			2-bromobenzoic acid	2.20		2.85		-1.95		0.48		1.82		-1.00		0.60		0.43
			W3			3-bromobenzoic acid	2.87		3.81		-0.32		1.46		2.76		-1.00		0.64		0.27
			W4			2-chlorobenzoic acid	2.05		2.94		-2.01		0.55		1.74		-1.00		0.70		0.43
			W5			4-chlorobenzoic acid	2.65		3.98		-0.37		1.34		2.64		-1.00		0.63		0.27
			W6			3-methylbenzoic acid	2.37		4.27		-0.36		1.10		2.21		-1.00		0.60		0.40
			W7			3,5-dimethylbenzoic acid	2.81		4.30		0.11		1.67		2.69		-1.00		0.59		0.38
			W8			phenylacetic acid	1.41		4.31		-1.28		0.77		1.79		-1.00		0.59		0.61
			W9			4-bromophenylacetic acid	2.31		4.19		-0.50		1.75		2.82		-1.00		0.61		0.56
			W10			2-chlorophenylacetic acid	2.10		4.07		-0.83		1.22		2.25		-1.00		0.61		0.55
			W11			2-methylphenylacetic acid	1.96		4.35		-0.69		1.16		2.15		-1.00		0.60		0.65
			W12			2-naphthalenecarboxylic acid	3.28		4.16		0.44		1.89		2.92		-1.00		0.65		0.46
			W13			1-naphthaleneacetic acid	2.74		4.24		-0.02		1.97		2.98		-1.00		0.60		0.67
			W14			2-chlorophenol	2.15		8.35		2.13		1.95		2.12		-0.03		0.32		0.31
			W15			4-bromophenol	2.59		9.31		2.59		2.37		2.52		0.00		0.67		0.20
			W16			2,6-dibromophenol	3.36		6.80		2.95		2.30		3.06		-0.55		0.47		0.22
			W17			2,4-dibromophenol	3.06		7.85		3.00		2.57		3.08		-0.15		0.49		0.27
			W18			2,4,6-trichlorophenol	3.69		6.21		2.83		2.74		3.73		-0.86		0.42		0.15
			W19			2,4,6-tribromophenol	4.33		5.95		3.24		3.13		4.26		-0.86		0.42		0.15
			W20			pentachlorophenol	4.69		5.12		2.80		3.82		4.93		-1.00		0.70		0.00
			W21			1,4-benzenedicarboxylic acid	NA		NA		/		/		1.27		-2.00		1.14		0.77
			W22			1,3,5-benzenetriol	NA		NA		/		0.23		0.96		-0.01		1.40		0.82
			W23			2-amino-5-nitrophenol	NA		NA		/		1.15		1.66		-0.04		0.76		0.64
			W24			1,4-benzenediol	NA		NA		/		0.21		0.60		0.00		1.06		0.57
			W25			2-amino-4-nitrophenol	NA		NA		/		0.74		1.86		-0.04		1.01		0.43
Compound type	No.			Compound				log P		pK_a_		log D_7.0_		log k_w-IS_		log k_w-IP_		n_e_		A	B
	W26			5-nitroisophthalic acid				NA		NA		/		2.89		2.86		-2.00		1.25	0.87
	W27			4-aminobenzoic acid				NA		NA		/		/		0.45		-0.99		0.80	0.76
	W28			2-aminophenol				NA		NA		/		0.65		0.78		0.00		0.60	0.66
	W29			4-hydroxyphenethyl alcohol				NA		NA		/		1.09		1.06		0.00		0.81	0.67
	W30			3-aminophenol				NA		NA		/		0.33		0.59		0.00		0.65	0.78
	W31			ethyl gallate				NA		NA		/		1.26		2.98		-0.15		1.35	0.93
	W32			2-amino-4-tert-butylphenol				NA		NA		/		2.58		2.68		0.00		0.51	0.59
	W33			3,5-dihydroxybenzoic acid				NA		NA		/		/		1.01		-1.00		1.56	0.93
Sulfonic acids	S1			benzenesulfonic acid				NA		NA		/		0.30		1.70		-1.00		0.31	0.88
	S2			1,5-naphthalenedisulfonic acid				NA		NA		/		/		2.30		-2.00		0.63	1.71
	S3			4-chlorobenzenesulfonic acid				NA		NA		/		1.27		2.57		-1.00		0.31	0.87
	S4			4-methylbenzenesulfonic acid				NA		NA		/		1.04		2.11		-1.00		0.31	0.88
	S5			5-amino-2-nanphthalenesulfonic acid				NA		NA		/		0.94		2.09		-1.00		0.54	1.26
	S6			2-amino-1,4-benzenedisulfonic acid				NA		NA		/		/		1.75		-2.00		0.85	1.90
	S7			1-naphthalenesulfonic acid				NA		NA		/		1.66		2.95		-1.00		0.31	0.94
	S8			2-naphthalenesulfonic acid				NA		NA		/		1.65		2.86		-1.00		0.31	0.94
	S9			2,4-dimethylbenzenesulfonic acid				NA		NA		/		1.28		2.64		-1.00		0.31	0.89
	S10			4-sulfobenzoic acid				NA		NA		/		/		1.54		-2.00		0.88	1.21
	S11			3,5-dichloro-2-hydroxybenzenesulfonic acid				NA		NA		/		1.95		3.75		-1.14		0.81	0.90
	S12			3,5-dicarbomethoxybenzenesulfonic acid				NA		NA		/		1.63		2.90		-1.00		0.31	1.55
	S13			4-hydroxybenzenesulfonic acid				NA		NA		/		/		1.15		-1.01		0.81	1.15
	S14			3-sulfobenzoic acid				NA		NA		/		/		1.85		-2.00		0.88	1.21

The *n*-octanol/water partition coefficients (log *P*) and p*K*_a_ were literature values obtained from the database module of ACD/Labs software; log *D*_7.0_ (log *D* under pH 7.0) were calculated with log *P*, p*K*_a_ and pH; log *k*_w-IS_ was log *k*_w_ value in IS-RPLC mode extrapolated by linear solvent strength (LSS) model; log *k*_w-IP_ was log *k*_w_ value in IP-RPLC mode extrapolated by LSS model; *n*_e_, *A* and *B* values were obtained from https://ilab.acdlabs.com/iLab2/; NA: no literature log *P*, p*K*_a_ value available; /: not available.

### 1.2 仪器与设备

实验所用高效液相色谱仪为Waters Alliance 2695(Waters, USA),配有真空脱气机、四元梯度泵、自动进样器、柱温箱和紫外-可见二极管阵列(PDA)检测器。数据的采集和处理均在Waters Empower色谱管理系统中进行。

pH值的测量使用SevenMulti型pH/电导率/离子综合测试仪(Mettler-Toledo, Switzerland),校准溶液为pH 4.01、7.02和9.26(25 ℃)的标准缓冲溶液(Mettler-Toledo)。

### 1.3 色谱条件

实验所用色谱柱为Welch Ultimate^®^ XB-C18(150 mm×4.6 mm, 5 μm,月旭科技(上海)股份有限公司),硅胶基质,孔径12 nm,比表面积320 m^2^/g,载碳量17%,封尾,耐受pH为1.5~10.0。实验中柱温始终保持在30 ℃,流速为1.0 mL/min,等度洗脱,每个化合物在最佳吸收波长处检测。实验中各化合物的进样浓度均为50 mg/L(甲醇配制),进样量为5 μL。

在本实验中,流动相的pH测定采用的方法是 ${}_{w}^{w}\mathrm{pH}$ ,^[18]^即在与有机调节剂混合之前,以水溶液作为pH电极的参比溶液,单独测定水相流动相的pH值。IS-RPLC流动相:甲醇-20 mmol/L磷酸二氢铵缓冲溶液(pH 7.0); IP-RPLC流动相:(甲醇+10 mmol/L四丁基溴化铵)-(20 mmol/L磷酸二氢铵+10 mmol/L四丁基溴化铵,pH 7.0)。

### 1.4 实验方法

在IS-RPLC和IP-RPLC色谱模式下,分别测定各化合物的保留时间(*t*_R_),使用尿嘧啶测定死时间(*t*_0_)。根据化合物疏水性的差异,每个化合物的*t*_R_至少在4个不同的甲醇比例下(*φ*=80%~10%,间隔10%或5%, *φ*是流动相中甲醇的体积分数)测定,*t*_R_使用双点校正法(DP-RTC)进行校正^[19,20]^。实验中所有化合物的*t*_R_均为至少3次独立进样的平均值。

保留因子*k*由公式*k*=(*t*_R_-*t*_0_)/*t*_R_计算得到,然后通过线性溶剂强度(LSS)模型(log *k*=log *k*_w_-*Sφ*,其中,*φ*是流动相中有机调节剂的体积分数,*S*是线性回归得到的常数)获取每个溶质的log *k*_w_,随后建立log *D*-log *k*_w_模型。采用Origin 9.4拟合模型并进行相关数据分析。

## 2 结果与讨论

### 2.1 log *k*_w_值的获取

分别建立每个化合物的log *k*-*φ*线性关系,外推获得log *k*_w_值,IS-RPLC模式下记为log *k*_w-IS_, IP-RPLC模式下记为log *k*_w-IP_。我们在前期工作^[21]^中已证明,中性化合物的保留时间几乎不受离子对试剂加入的影响,且由于中性化合物的疏水性较大,往往需要在较高的有机调节剂比例下获得合适的保留,而在含盐的高比例有机调节剂下再加入离子对试剂,更容易造成盐的析出从而堵塞色谱柱和仪器,因此,对于中性化合物,我们只测定了IS-RPLC模式下的*t*_R_值,并以IS-RPLC模式下推导的log *k*_w-IS_实验值代表了IP-RPLC模式下的log *k*_w-IP_值,即log *k*_w-IS_等于log *k*_w-IP_。在未加入离子对试剂的条件下,强离解化合物(W21、W27、W33、S2、S6、S10、S13、S14)在色谱柱上的保留极弱,即使在*φ*=10%时,保留时间依然小于死时间,因此,无法获取它们的log *k*_w-IS_。当加入离子对试剂后,大部分离解化合物尤其强离解化合物的保留均得到明显增强。log *k*-*φ*关系显示,所有化合物的线性相关系数(*R*^2^)均大于0.99,表明在硅胶基质C18柱上,中性、弱离解化合物及强离解化合物的保留行为均符合LSS模型。部分弱离解和强离解化合物在离子对试剂加入前后的log *k*-*φ*关系如图1所示,各化合物的log *k*_w-IS_和log *k*_w-IP_列于表1。

**图1:**
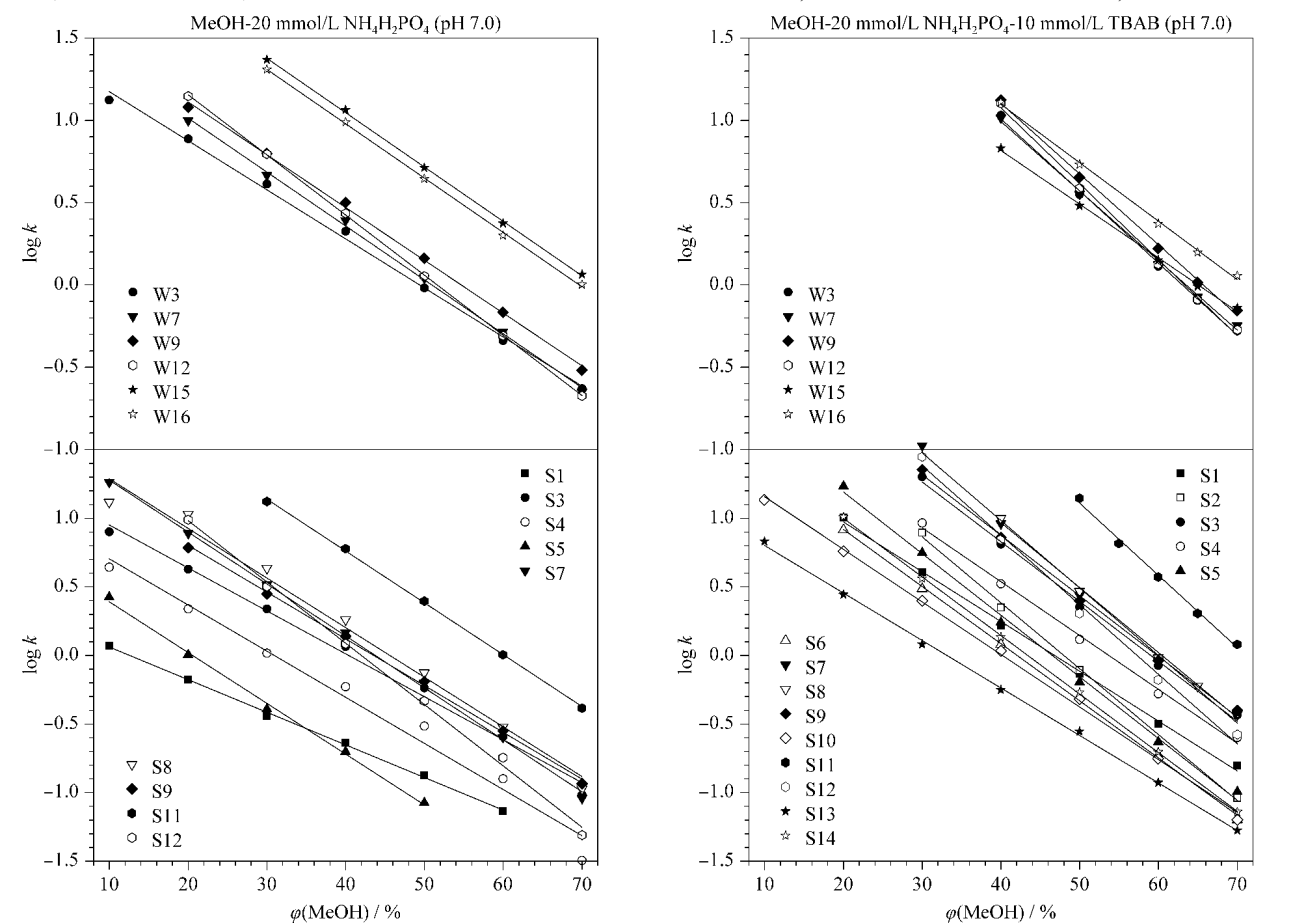
部分弱离解和强离解化合物在不同流动相下的log *k*-*φ*关系

### 2.2 log *D*-log *k*_w-IS_模型

中性化合物的log *D*_7.0_值(pH 7.0条件下的log *D*值)等于其log *P*值。对于有log *P*和p*K*_a_文献实验值的弱离解酸性化合物,其log *D*_7.0_值由其log *P*、p*K*_a_以及流动相pH计算获取^[22]^,结果列于表1。部分无log *D*_7.0_值的离解化合物(如W22~26、W29~32、S1、S3等)在IS-RPLC模式下有明显保留,根据LSS模型能够获取其log *k*_w-IS_值(见表1),因此,可以基于log *D*_7.0_-log *k*_w-IS_模型测定这部分化合物的log *D*_7.0_值。在IS-RPLC模式下,我们以26种中性化合物(N1~26)和20种弱离解酸性化合物(W1~20)为模型化合物,首先在不引入其他参数的情况下,建立log *D*_7.0_-log *k*_w-IS_模型:


(1) log *D*_7.0_=-0.072(±0.022) log *k*_w-IS_+ 3.928(±0.596) *N*=46, *R*^2^=0.1745

其中,*N*为模型化合物的个数。可以看出,模型(1)的线性极差,说明除疏水作用外,还存在着其他次级作用影响溶质的保留。因此,我们尝试在log *D*_7.0_-log *k*_w-IS_模型中分别引入氢键参数*A*和*B*以及静电荷*n*_e_(列于表1),分别建立相应的模型:


(2) log *D*_7.0_=1.251(±0.08) log *k*_w-IS_- 1.790(±0.40)*A*-0.630(±0.32) *N*=46, *R*^2^=0.9254

(3) log *D*_7.0_=1.360(±0.105) log *k*_w-IS_- 1.271(±0.684)*B*-1.054(±0.446) *N*=46, *R*^2^=0.8993

(4) log *D*_7.0_=1.205(±0.064) log *k*_w-IS_+ 1.420(±0.190)*n*_e_-0.480(±0.232) *N*=46, *R*^2^=0.9525

显然,引入氢键参数*A*或*B*后,模型的线性相关性均得到了极大地改善,引入*n*_e_后模型的改善更为明显(*R*^2^=0.9525)。这是由于在pH 7.0的条件下,模型中所用弱酸性化合物大多数处于离解状态,且离解状态不同,而此时硅胶基质色谱柱上的硅羟基部分解离,因此,溶质与固定相之间会表现出较强的氢键相互作用和静电相互作用。通过继续优化模型发现,将*n*_e_、*A*和*B*同时引入后的模型(5)相关性最佳且均方差最小,模型如下:


(5) log *D*_7.0_=1.097(±0.077) log *k*_w-IS_+ 1.648(±0.293)*n*_e_+0.524(±0.500)*A*- 1.236(±0.448) *B*+0.090(±0.332) *N*=46, *R*^2^=0.9583

随后,我们选用模型(5)预测了在IS-RPLC模式下有保留的19种离解化合物的log *D*_7.0_值,结果列入表2中,测定的数据将用于后续IP-RPLC模型的建立和模型的验证。

**表2:** 待测化合物的log *D*_7.0_实验预测值

No.	Compound	log D_7.0_		Error^(d)^
		ACD/Labs^(a)^	Predicted in this work	
W22	1, 3,5-benzenetriol	0	0.04^(b)^	0.04
W23	2-amino-5-nitrophenol	1.13	0.90^(b)^	-0.23
W24	1,4-benzenediol	0.62	0.17^(b)^	-0.45
W25	2-amino-4-nitrophenol	0.66	0.84^(b)^	0.18
W26	5-nitroisophthalic acid	-2.80	-0.46^(b)^	2.34
W28	2-aminophenol	0.49	0.30^(b)^	-0.19
W29	4-hydroxyphenethyl alcohol	0.85	0.88^(b)^	0.03
W30	3-aminophenol	0.61	-0.18^(b)^	-0.79
W31	ethyl gallate	1.22	0.79^(b)^	-0.43
W32	2-amino-4-tert-butylphenol	2.27	2.46^(b)^	0.19
S1	benzenesulfonic acid	-4.05	-2.16^(b)^	1.89
S3	4-chlorobenzenesulfonic acid	-3.34	-1.08^(b)^	2.26
S4	4-methylbenzenesulfonic acid	-2.57	-1.34^(b)^	1.23
S5	5-amino-2-nanphthalenesulfonic acid	-3.91	-1.81^(b)^	2.10
S7	1-naphthalenesulfonic acid	-2.87	-0.74^(b)^	2.13
S8	2-naphthalenesulfonic acid	-2.87	-0.75^(b)^	2.12
S9	2,4-dimethylbenzenesulfonic acid	-2.18	-1.09^(b)^	1.09
S11	3,5-dichloro-2-hydroxybenzenesulfonic acid	-3.15	-0.33^(b)^	2.82
S12	3,5-dicarbomethoxybenzenesulfonic acid	-3.23	-1.53^(b)^	1.70
W21	1,4-benzenedicarboxylic acid	-2.13	-4.28^(c)^	-2.15
W27	4-aminobenzoic acid	-1.41	-2.79^(c)^	1.38
W33	3,5-dihydroxybenzoic acid	-2.10	-2.39^(c)^	-0.29
S2	1,5-naphthalenedisulfonic acid	-6.60	-4.69^(c)^	1.91
S6	2-amino-1,4-benzenedisulfonic acid	-7.73	-5.55^(c)^	2.18
S10	4-sulfobenzoic acid	-5.54	-4.70^(c)^	0.84
S13	4-hydroxybenzenesulfonic acid	-5.14	-2.70^(c)^	2.44
S14	3-sulfobenzoic acid	-5.36	-4.36^(c)^	1.00

(a) calculated using ACD/Labs software V11.02 (© 1994-2021 ACD/Labs); (b)predicted by model (5) using IS-RPLC method; (c) predicted by model (6) using IP-RPLC method; (d) log *D*_7.0_ predicted in this work-log *D*_7.0_ calculated using ACD/Labs.

### 2.3 log *D*-log *k*_w-IP_模型的建立及强离解化合物log *D*的测定

Liang等^[17]^的研究表明,在IP-RPLC模式下,通过引入*n*_e_、*A*和*B*可以将中性化合物、弱离解以及强离解酸性化合物联系起来建立测定强离解化合物log *D*值的模型。基于此,我们以中性化合物N1~26、弱离解酸性化合物W2~19以及由IS-RPLC测得log *D*_7.0_值的离解化合物W22~26、W28~32、S1、S3~5、S7~9、S11共同作为混合模型组,建立IP-RPLC模式下log *D*_7.0_测定的多元线性模型(6):


(6) log *D*_7.0_=1.075(±0.077) log *k*_w-IP_+ 2.366(±0.166)*n*_e_+0.120(±0.245)*A*- 1.554(±0.320)*B*+0.150(±0.310) *N*=62, *R*^2^=0.9425

可以看出,模型(6)的*R*^2^可以达到0.94,说明模型具有良好的线性相关性,充分证明在硅胶基质C18色谱柱上以不同类型化合物作为模型化合物建立log *D*-log *k*_w-IP_模型的可行性。Liang等^[16]^在聚乙烯醇基质C18柱上得到的多元线性模型如下:


(7) log *D*_7.0_=1.18(±0.07) log *k*_w-IP_+ 1.55(±0.26)*n*_e_-0.09(±0.44)*A*- 0.44(±0.26)*B*-0.82(±0.31) *N*=50, *R*^2^=0.954

对比发现,本工作中在硅胶基质C18柱上模型的相关性与Liang等在聚乙烯醇基质C18柱上模型的相关性非常接近。同时发现,本工作模型中log *k*_w-IP_的系数更接近于1,而*n*_e_、*A*和*B* 3个参数的系数均大于在聚乙烯醇基质C18柱上得到的模型系数,尤其是*n*_e_的系数,这表明在硅胶基质C18柱上存在着更强的次级作用,参数*n*_e_、*A*和*B*对模型的贡献更大。

为了验证模型的可靠性,我们选取3种不同类型的离解化合物作为验证化合物,分别为五氯酚(W20)、苯甲酸(W1)和3,5-二(甲氧基羰基)苯磺酸(S12),其中3,5-二(甲氧基羰基)苯磺酸无SFM/SSM文献log *D*_7.0_值,其log *D*_7.0_标准值采用模型(5)得到的测定值。将3个验证化合物的log *k*_w-IP_、*n*_e_、*A*和*B*带入模型(6),测定其log *D*_7.0_,结果见表3。

**表3:** log *D*-log *k*_w-IP_模型的外部验证

Compound	log D_7.0_				Errors	
	SFM/SSM^(a)^	IP-RPLC^(c)^	ACD/Labs^(d)^		1	2
Pentachlorophenol	2.80	2.52	3.20			
Benzoic acid	-0.93	-0.97	-1.12		-0.04	-0.15
3,5-Dicarbomethoxybenzenesulfonic acid	-1.53^(b)^	-1.47	-3.23		0.06	-1.70

(a) literature SFM/SSM data obtained from database module of ACD/Labs software; (b) predicted by model (5) using IS-RPLC method in this work; (c) predicted by model (6) using IP-RPLC method in this work; (d) calculated values using ACD/Labs software V11.02 (© 1994-2021 ACD/Labs). Errors: 1. log *D*_7.0_ (IP-RPLC)-log *D*_7.0_ (SFM/SSM); 2. log *D*_7.0_ (ACD/Labs)-log *D*_7.0_ (SFM/SSM).

从表3中可以看出,用模型(6)预测的3种离解化合物的log *D*_7.0_与SFM/SSM法(苯甲酸和五氯酚)或者本文IS-RPLC法(3,5-二(甲氧基羰基)苯磺酸)测定得到的log *D*_7.0_非常相近,误差在10%以内,说明所建立模型既能测定弱离解化合物的log *D*值也能测定强离解化合物的log *D*值,充分证明了模型的可靠性。ACD/Labs软件是公认的最准确的log *P*和log *D*计算软件,由表3可见,与标准值相比,当化合物结构较为简单时,如五氯酚和苯甲酸,用ACD/Labs软件计算得到的log *D*值比较准确,但是当待测化合物的结构稍复杂时,如3,5-二(甲氧基羰基)苯磺酸,由ACD/Labs软件计算得的log *D*值存在较大的误差,与俞慧敏等^[23]^的发现一致。

利用模型(6)预测了对苯二甲酸、对氨基苯甲酸、3,5-二羟基苯甲酸、1,5-萘二磺酸、苯胺-2,5-二磺酸、4-磺基苯甲酸、4-羟基苯磺酸和3-磺基苯甲酸的log *D*_7.0_值,结果一并列于表3中。结果显示,对这类离解性强、结构较为复杂的化合物,其log *D*_7.0_值与ACD/Labs软件计算得到的值之间差别较大,也说明用ACD/Labs软件计算强极性化合物和结构复杂的化合物时需要谨慎。

## 3 结论

本文在硅胶基质C18色谱柱上分别研究了中性、弱离解和强离解化合物在IS-RPLC和IP-RPLC中的保留行为。研究发现,IS-RPLC模式下,当模型化合物的离解不能被完全抑制时,引入静电荷*n*_e_、氢键参数*A*和*B*可以有效改善模型的相关性。更为重要的是,在IP-RPLC模式下,我们通过扩大模型化合物的范围,证明了在硅胶基质C18柱上测定强离解化合物log *D*的可行性,证实了本课题组Liang等提出的利用不同类型化合物作为模型化合物构建QSRR模型用于测定强离解化合物log *D*值的方案在不同基质色谱柱上的通用性。
